# Recovery from indomethacin-induced gastrointestinal bleeding by treatment with teprenone

**DOI:** 10.1186/s40780-023-00312-y

**Published:** 2023-11-28

**Authors:** Saori Deguchi, Ayusa Iwakami, Mizuki Tujigiwa, Hiroko Otake, Yu Mano, Naoki Yamamoto, Yosuke Nakazawa, Manju Misra, Noriaki Nagai

**Affiliations:** 1 Faculty of Pharmacy, Kindai University, 3-4-1 Kowakae, Higashi-Osaka,Osaka, 577-8502 Japan; 2 Department of Pharmacy, Bell Land General Hospital, 500-3, Higashiyama, Naka-ku, Sakai, Osaka 599-8247 Japan; 3 Support Office for Bioresource Research, Research Promotion Headquarters, Fujita Health University, 1-98 Dengakugakubo, Kutsukake-cho, Toyoake, Aichi 470-1192 Japan; 4 Faculty of Pharmacy, Keio University, 1-5-30 Shibakoen, Minato-ku, Tokyo, 105-8512 Japan; 5 Department of Pharmaceutics, National Institute of Pharmaceutical Education and Research; Opposite AirForce Station, Palaj Basan Road, Village Palaj, Gandhinagar, 382355 Gujarat India; 6 Graduate school of Pharmacy, Gujarat Technological University Gandhinagar Campus Nr. Government Polytechnic K-6 Circle, E-4 Electronic Estate G.I.D.C, Sector-26, Gandhinagar, 382028 Gujarat India

**Keywords:** Gastrointestinal injury, Teprenone, Indomethacin, Rheumatoid arthritis, Nitric oxide

## Abstract

**Background:**

Gastrointestinal injuries caused by nonsteroidal anti-inflammatory drugs (NSAIDs) is a serious side effect in patients with rheumatoid arthritis (RA). However, effective therapeutic strategies have yet to be established. In this study, we investigated the therapeutic effects of teprenone (TEP), a gastric mucosal protective drug, on NSAID-induced gastrointestinal injuries in rats with RA (AA rats).

**Methods:**

Gastrointestinal injury was induced by oral administration of indomethacin (IMC), a typical NSAID. TEP was orally administered after IMC-induced gastrointestinal bleeding, and the stomach, jejunum, and ileum were excised.

**Results:**

On day 14 of IMC administration, lesion areas in the stomach, jejunum, and ileum were significantly larger in AA rats than in normal rats. When TEP was orally administered to AA rats, the lesion areas in the stomach, jejunum, and ileum significantly decreased compared with those in control rats (IMC-induced AA rats). Therefore, we measured NOS2 mRNA and NO levels, which were significantly decreased in rats with IMC-induced AA after treatment with TEP.

**Conclusions:**

These results suggest that the oral administration of TEP may be useful for the treatment of NSAID-induced gastrointestinal injuries in patients with RA.

## Background

Rheumatoid arthritis (RA) is a chronic inflammatory disease that mainly affects the joints [1]. The release of inflammatory cytokines and the overproduction of nitric oxide (NO) cause synovial inflammation and bone destruction in RA [2], which are primarily treated with drugs. Nonsteroidal anti-inflammatory drugs (NSAIDs) are used at the beginning of treatment to relieve the pain caused by RA [3]. The use of NSAIDs is essential for the treatment of RA; however, side effects, such as gastrointestinal injuries caused by NSAIDs hinder the treatment of RA in severe cases [4]. It has been reported that the prevalence of intestinal lesions caused by NSAIDs in patients without RA were 11.9%, whereas that in RA patients were 56.8% [5]. NSAIDs inhibit cyclooxygenase and reduce prostaglandin (PG) production, which acts as a defense factor in the gastric mucosa. Moreover, direct stimulation with NSAIDs produces excessive NO *via* inducible NO synthases (iNOS, NOS2), resulting in gastrointestinal injury [6]. In addition, patients with RA are more likely to develop gastrointestinal injuries following the oral administration of NSAIDs because of overproduction of NOS2-derived NO [7].

In general, if side effects occur, NSAIDs can be discontinued, or the patient can be treated with a proton pump inhibitor (PPI) or PG preparation [8]. However, we previously reported that the oral administration of rebamipide (RBM) enhanced the repair of indomethacin (IMC)-induced gastrointestinal injuries in adjuvant-induced arthritis (AA) rats, an animal model of RA [9]. Therefore, gastrointestinal injuries caused by NSAIDs may be treated with mucosal protective drugs such as RBM and teprenone (TEP) [10–12]. However, to the best of our knowledge, there are no reports that oral administration of TEP is effective in treating NSAID-induced gastrointestinal injuries in patients with RA, and TEP is currently not prescribed for NSAID-induced gastrointestinal injuries.

TEP, 6,10,14,18-tetramethyl-5,9,13,17-nonadecatetraen-2-one [a mixture of (5*E*,9*E*,13*E*)- and (5*Z*,9*E*,13*E*)- isomers], is used to treat gastritis and gastric ulcers. In addition to promoting gastric mucus secretion and increasing gastric blood flow, TEP exerts a protective effect on the gastric mucosa by reducing myeloperoxidase activity in neutrophils. In addition, TEP regenerates the mucosal epithelium by increasing the levels of hexosamines, which are proteins found in the mucus [13–15]. In this study, we investigated whether TEP promotes the repair of NSAID-induced gastrointestinal injury in AA rats.

## Methods

### Chemicals

IMC was obtained from Wako Pure Chemical Industries, Ltd. (Osaka, Japan). TEP was prepared by Tokyo Chemical Industry Co., Ltd. (Saitama, Japan), and 2-hydroxypropyl-β-cyclodextrin (HPβCD) was purchased from Nihon Shokuhin Kako Co., Ltd. (Tokyo, Japan). Indomethacin, diethyl ether, and Bayol F were purchased from Wako Pure Chemical Industries, Ltd. (Osaka, Japan). Carboxymethyl cellulose was obtained from Hirai Chemical Co., Ltd. (Osaka, Japan). *Mycobacterium butyricum* was purchased from Becton Dickinson and Company, Ltd. (Fukushima, Japan). Saline was purchased from Otsuka Pharmaceutical Co. Ltd. (Tokyo, Japan).

### Animals

Male 7-week-old DA rats (weighing approximately 150 g) were obtained from Shimizu Laboratory Supplies Co., Ltd. (Kyoto, Japan). The animals were housed at 25°C (room temperature) and were fed a commercial diet (CE-2; Clea Japan Inc., Tokyo, Japan) and water ad libitum. The experimental protocol was approved by the Kindai University (project identification code KAS-2022-010). This study was performed in accordance with the guidelines of the Japanese Pharmacological Society and the International Association for the Study. Rat health and behavior were monitored daily. Adjuvant injections were administered under anesthesia to minimize distress. Euthanasia was performed based on the duration of the experiment, because none of the models showed a significant reduction in the humane endpoints. At 7, 14, 21, and 42 days after the adjuvant injection, the rats were euthanized to measure paw edema and gastrointestinal lesion levels. Arthritic paw edema was assessed by measuring the paw volume using a plethysmometer. In addition, IMC was administered 14 days after adjuvant injection to rats, and the stomach was removed 24 h later (TEP treatment; 6 h after TEP administration, 18 h later) or 48 h later (TEP treatment; TEP was administered 24 h later, and 24 h later), the jejunum and ileum were removed, and the experiment was terminated. Euthanasia was performed by assisted exsanguination under deep anesthesia according to the AVMA guidelines 2020 [16].

### Induction of arthritis in DA Rats

*Mycobacterium butyricum* (100 mg) was polished using an agate mortar containing of Bayol F oil (10 mL). It was then subdivided into plastic cryogenic vials, sterilized in an autoclave (120°C, 15 min), and stored at 4°C (cold temperature). The prepared solution was administered through a needle (27G) into the right hind leg and tail of the rats (50 μL). Fourteen days later, the legs reached their maximum volume, and the rats were used as models of arthritis (AA rats).

### Evaluation of gastrointestinal bleeding

**Fig. 1:**
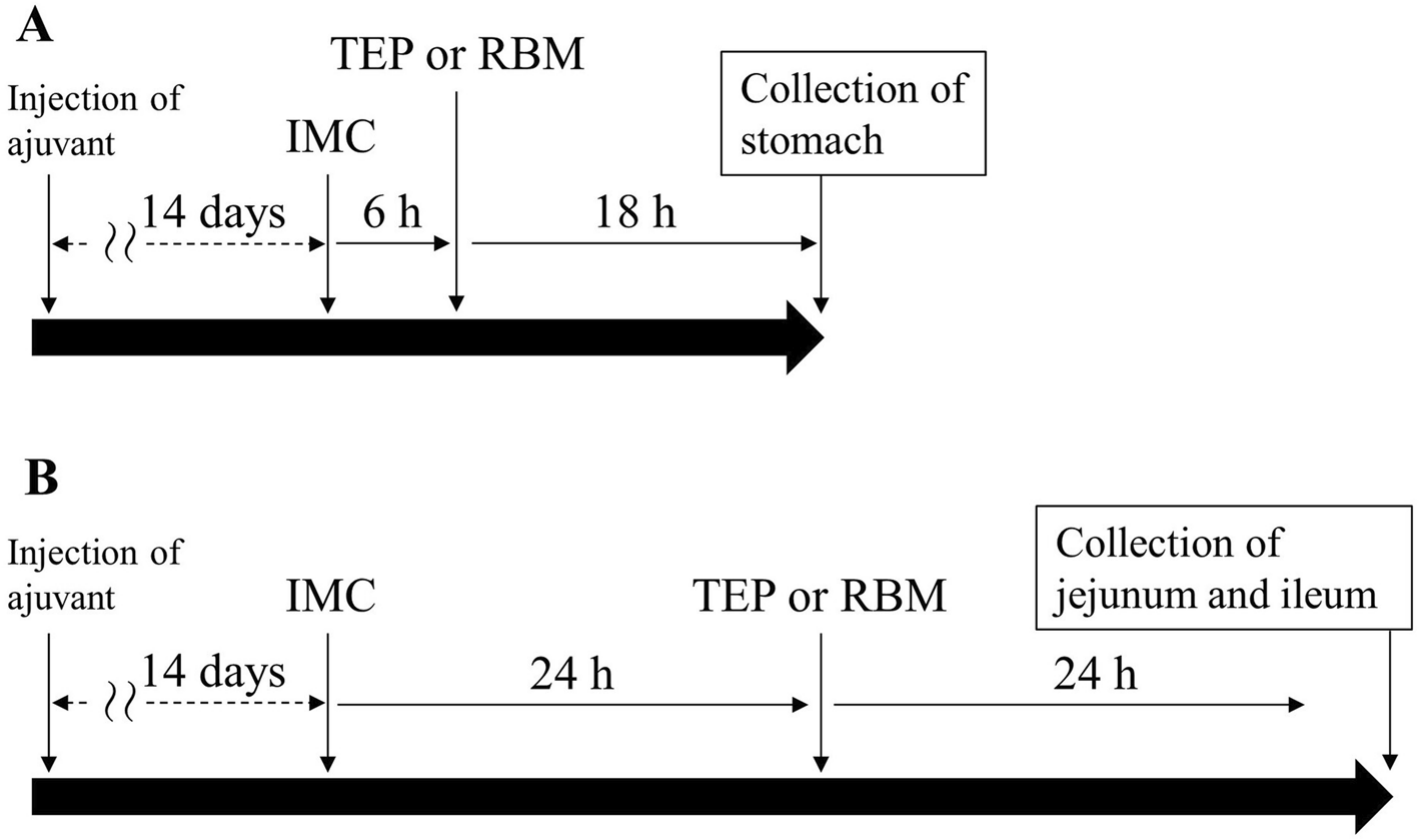
Protocol of TEP administration in IMC-Induced Gastric Ulcerogenic (**A**) and Intestinal Ulcerogenic Lesions (**B**) of AA rats

Gastrointestinal bleeding was induced by the oral administration of IMC (40 mg/kg) diluted in 1% carboxymethyl cellulose to normal and AA rats that had fasted since the previous day. After 24 h, the stomach was removed, and the jejunum and ileum were removed after 48 h. The organs (stomach, jejunum, and ileum) were rinsed thoroughly with saline and photographed using a digital camera. The areas of the black damaged parts, which are IMC-induced gastrointestinal injuries, were analyzed using ImageJ, and the provided values were converted to actual measurement values. The lesion area (%) was presented as the gastrointestinal bleeding area (black damaged parts)/total area (stomach, jejunum, or ileum) × 100. In this study, TEP (3 mg/kg) and RBM (2 mg/kg) were orally administered to investigate the recovery effect in IMC-induced gastrointestinal injuries, and the doses of TEP and RBM were determined based on the human dosing. Briefly, the powder of TEP (3 mg/mL) and RBM (2 mg/mL) were diluted in 5% HPβCD, and the suspensions (1 mL/kg) were orally administered 6 h after IMC-induced gastrointestinal bleeding, and stomachs were removed 18 h later. TEP was administered 24 h after IMC-induced gastrointestinal bleeding and the jejunum and ileum were removed 24 h later. Figure 1 shows the schedule of administration of IMC, TEP, and RBM.

### Quantitative PCR (Real-Time PCR)

Samples were analyzed by real-time PCR using a LightCycler^®^ 2.0 DX400 instrument (Roche Diagnostic Applied Science, Mannheim, Germany). Total RNA was extracted from stomach, jejunum, and ileum tissues using the acid guanidinium thiocyanate-phenol-chloroform extraction method according to the manufacturer’s instructions. PCR was conducted using an RNA PCR Kit (TaKaRa Bio Inc., Shiga, Japan) and LightCycler FastStart DNA Master SYBR Green I (Roche Diagnostics Applied Science, Mannheim, Germany), according to the manufacturer’s instructions. The primers used were glyceraldehyde-3-phosophate (GAPDH; 5′-ACGGCACAGTCAAGGCTGAGA-3′ and 5′-CGCTCCTGGAAGATGGTGAT-3′, NM_01708) or NOS2 (5′-GGAGAGATTTTTCACGACACCC-3′ and 5′-CCATGCATAATTTGGACTTGCA-3′, NM_012611). The PCR conditions were as follows: denaturation at 95°C for 10 sec, annealing at 60°C for 10 sec, and extension at 72°C for 5 sec. The NOS2 mRNA levels were calculated using the NOS2/GAPDH ratio.

### Measurement of NO levels

The stomach, jejunum, and ileum tissues were homogenized in saline on ice and the homogenates were centrifuged at 20,400×g for 20 min at 4°C. The NO levels in the supernatants were measured after centrifugation using ENO-20 (Eicom, Kyoto, Japan). Briefly, NO_2_^-^ and NO_3_^-^ were separated on a NO-PAK column (4.6×50 mm, Eicom, Kyoto, Japan). NO_2_^-^ was measured as the total NO level by Griess reagent using NOD-10 (Eicom, Kyoto, Japan) at 540 nm.

### Hematoxylin and Eosin (H&E) and immunohistochemical staining of the gastrointestinal injuries

After the animals were euthanized, their organs (stomach, jejunum, and ileum) were fixed with formalin. Paraffin sections were prepared from the fixed organs in the usual manner. During preprocessing, endogenous peroxidase was inactivated with 0.3% hydrogen peroxidase in methanol, and the samples were pretreated with a citrate buffer solution (pH 6.0, 90°C, 20 min). Sections were washed with PBS (Sigma-Aldrich, St. Louis, MO, USA) and nonspecifically blocked using Protein Block Serum-Free solution (Agilent Technologies, Santa Clara, CA, USA). Anti-rat osteopontin (OPN) rabbit monoclonal antibody was used as the primary antibody (1:50; Immuno-Biological Laboratories Co, Ltd, Gunma, Japan), and Histofine Simple Stain MAX-PO (Nichirei, Tokyo, Japan) was used as the secondary antibody. The reaction product was visualized using 3, 3’-diaminobenzidine tetrahydrochloride solution (DAB, Agilent Technologies), and the nuclei were counterstained with Mayer’s hematoxylin. Samples were analyzed using a microscope (Power BX-51; Olympus, Tokyo, Japan). Hematoxylin and eosin (H&E) staining was performed on the continuous sections.

### Statistical analysis

Data are expressed as the mean ± standard error of the mean (S.E.), and the number of animals studied is represented by n. For statistical analysis of multiple group comparisons, one-way repeated-measures ANOVA followed by Dunnett’s test was used. Statistical differences between the two groups were analyzed using the unpaired Student’s t-test. Statistical significance was considered at *P* < 0.05.

## Results

### Gastrointestinal ulcerogenic response to IMC in AA Rats

**Fig. 2:**
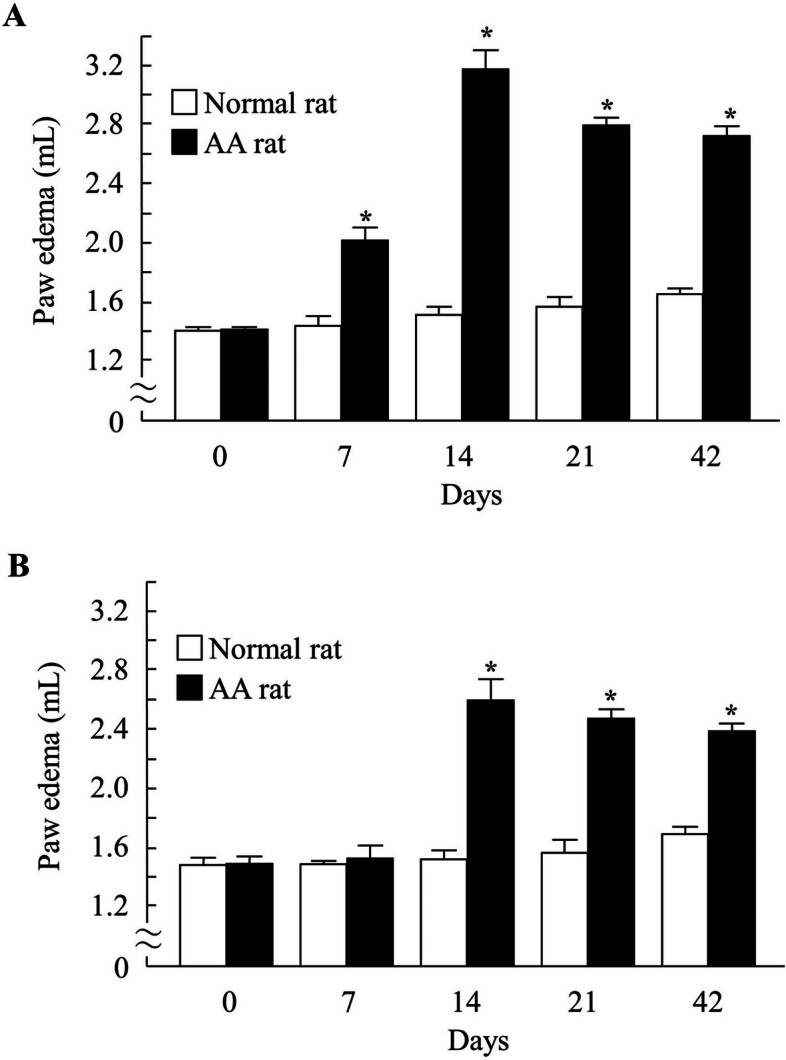
Paw Volume of the Right (**A**) and Left (**B**) Hind Feet of Normal and AA Rats 0–42 day after the Injection of Adjuvant. Open columns: normal rats; closed columns: AA rats. *n*=6–9. *P<0.05 vs normal rat for each category. The paw edema in the right and left hind feet of AA rats were observed at 7–42 day and 14–42 day, respectively, and the paw edema in the both of right and left find feet reached a maximum 14 day after injection

**Fig. 3:**
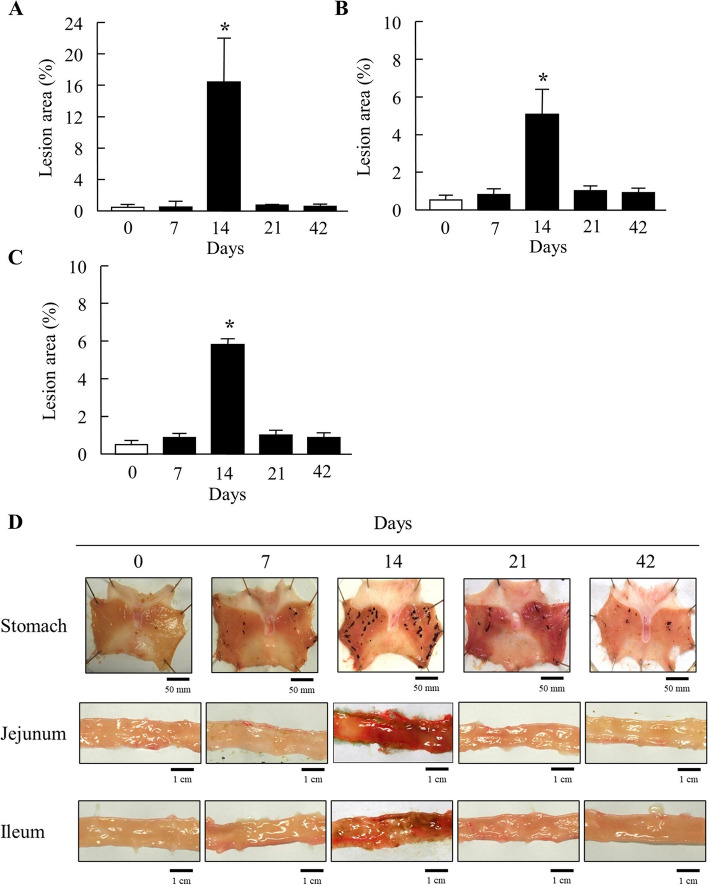
Changes in the Gastric and Intestinal Ulcerogenic Response to IMC in AA Rats. **A**–**C** lesion areas in the stomach (**A**), jejunum (**B**) and ileum (**C**) of IMC-treated AA rats. **D** images in the stomach, jejunum and ileum of IMC-treated AA rats. Open columns: normal rats; closed columns: AA rats. *n*=3–6. *P<0.05 vs normal rat for each category. The lesion area in the stomach, jejunum and ileum of AA rats were increased 14 day after the injection

Figure 2 shows the changes in paw volume in the right and left hind feet of the AA rats. Paw edema was observed after the administration of the adjuvant, which started on day 7 in the right hind feet of AA rats and peaked 14 days after injection. Subsequently, the paw edema persisted for 42 days. However, paw edema was not observed in the left hind foot until 14 days after adjuvant injection and was observed on days 21 and 42. Figure 3 shows the lesion areas in the stomach, jejunum, and ileum of IMC-treated AA rats 7–42 days after the adjuvant injection. The lesion areas in IMC-treated rats with AA at 7, 21, and 42 days after adjuvant injection were similar to those in IMC-treated normal rats. However, the lesion areas in IMC-treated AA rats 14 days after adjuvant injection were significantly larger than those in IMC-treated normal rats. Specifically, when IMC was administered to normal rats, the lesion area (%) into the stomach, jejunum, and ileum was 0.56 ± 0.29, 0.56 ± 0.18, and 0.51 ± 0.15, respectively (%, mean ± S.E., *n*=6–15). The lesion areas in the stomach, jejunum, and ileum of IMC-treated AA rats were 16.5-, 5.1-, and 5.9-fold higher, respectively, than those in normal rats.

### Recovery Effect of TEP on Gastric Lesions in IMC-Treated AA Rats

**Fig. 4:**
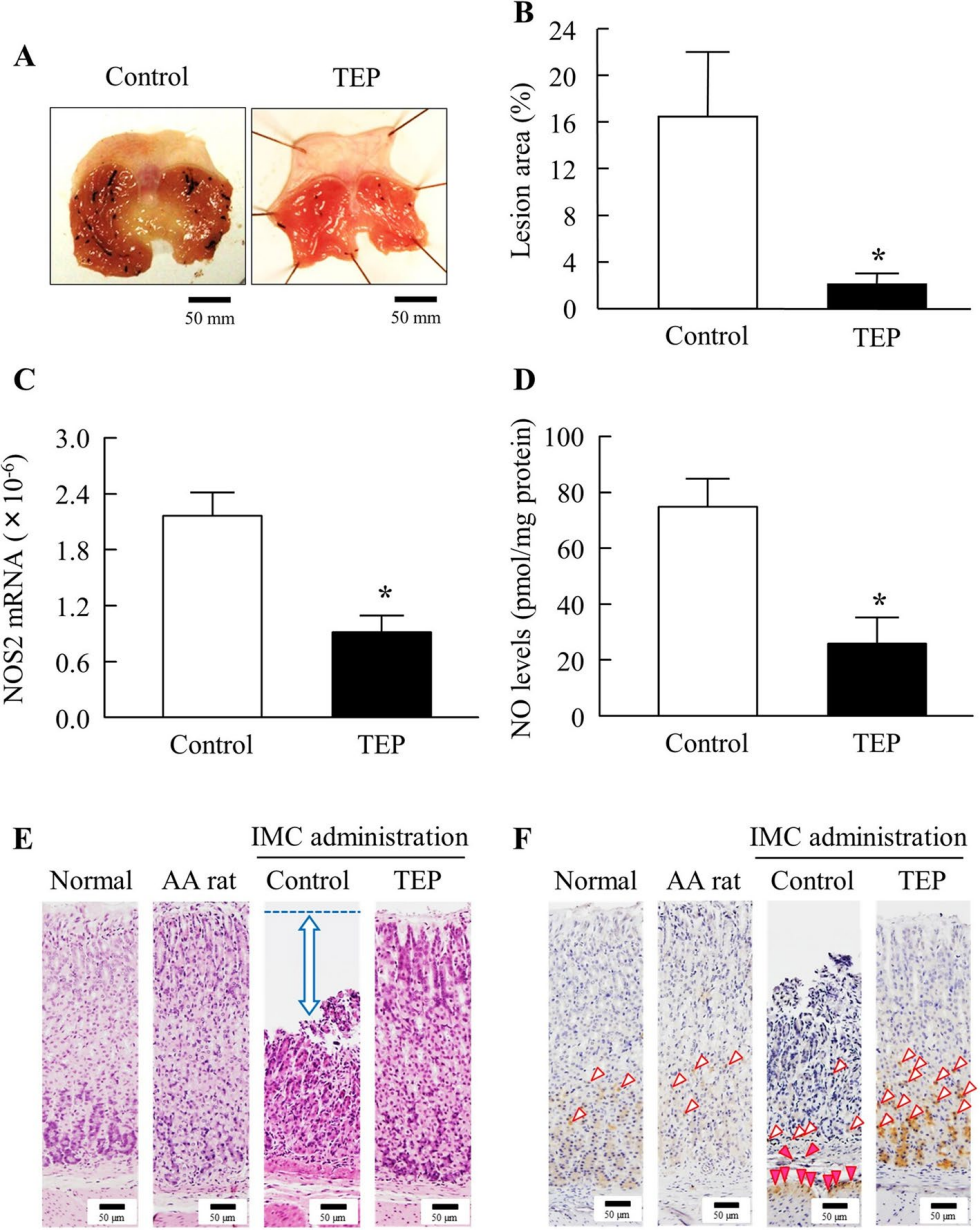
Effect of the TEP on IMC-Induced Gastric Ulcerogenic Lesions in AA Rats 14 day after Adjuvant Injection. **A** images of the stomach of IMC-treated AA rats treated with TEP. **B**–**D** effect of TEP on lesion area, NOS2 mRNA and NO levels in the stomach of IMC-treated AA rats. **E** and **F** image of H&E-stained **E** and OPN immunostained stomach tissue of IMC-treated AA rats treated with TEP. Both arrows, areas of desquamation. Open arrowheads, OPN immunostained-positive cells in the mucosal layer. Closed arrowheads, OPN immunostained-positive cells in the submucosal and muscular layers. Bars in the image indicate 50 µm. *n*=3–9. *P<0.05 vs control for each category. TEP attenuated the excessive NO *via* NOS2 levels, and promote recovery from IMC-induced gastric ulcerogenic lesions in AA rats

Figure 4A and B show the changes in gastric lesions in IMC-treated AA rats, with or without TEP treatment. The IMC-induced gastric lesions significantly improved after TEP treatment. Moreover, changes in NOS2 mRNA and NO levels in IMC-treated AA rats, with or without TEP treatment, were investigated (Fig. 4C and D). TEP attenuated both NOS2 mRNA and NO levels; the NOS2 mRNA and NO levels in IMC-treated AA rats treated with TEP were 0.4- and 0.3-fold, respectively, compared to those in the control group. Moreover, NOS2 mRNA of normal rats were 0.40 ± 0.24 (×10^-6^), and that of AA rats were 0.41 ± 0.11 (×10^-6^). NO levels of normal rats were 8.9 ± 4.2 (pmol/mg protein), and that of AA rats were 10.7 ± 2.4 (pmol/mg protein) (*n*=6). Figure 4E and F show tissue specimens from the stomachs of normal rats, AA rats and IMC-treated AA rats, with or without TEP treatment. In H&E-stained tissue specimens, no differences were observed in the structure of the mucosal layers of normal, AA, and TEP-treated AA rats. However, in the areas of IMC-treated AA rats without TEP treatment (control group), superficial mucosal cell shedding was observed (both arrows). In the mucosal layer, OPN-positive cells were observed in the gastric glands of normal, AA, and TEP-treated AA rats. However, in OPN immunostaining of the control group, positive cells were observed in the submucosal and muscular layers. In OPN immunostaining, the mucus within the glandular lumen of the gastric glands is nonspecific.

### Recovery effect of TEP on intestinal lesions in IMC-treated AA Rats

**Fig. 5:**
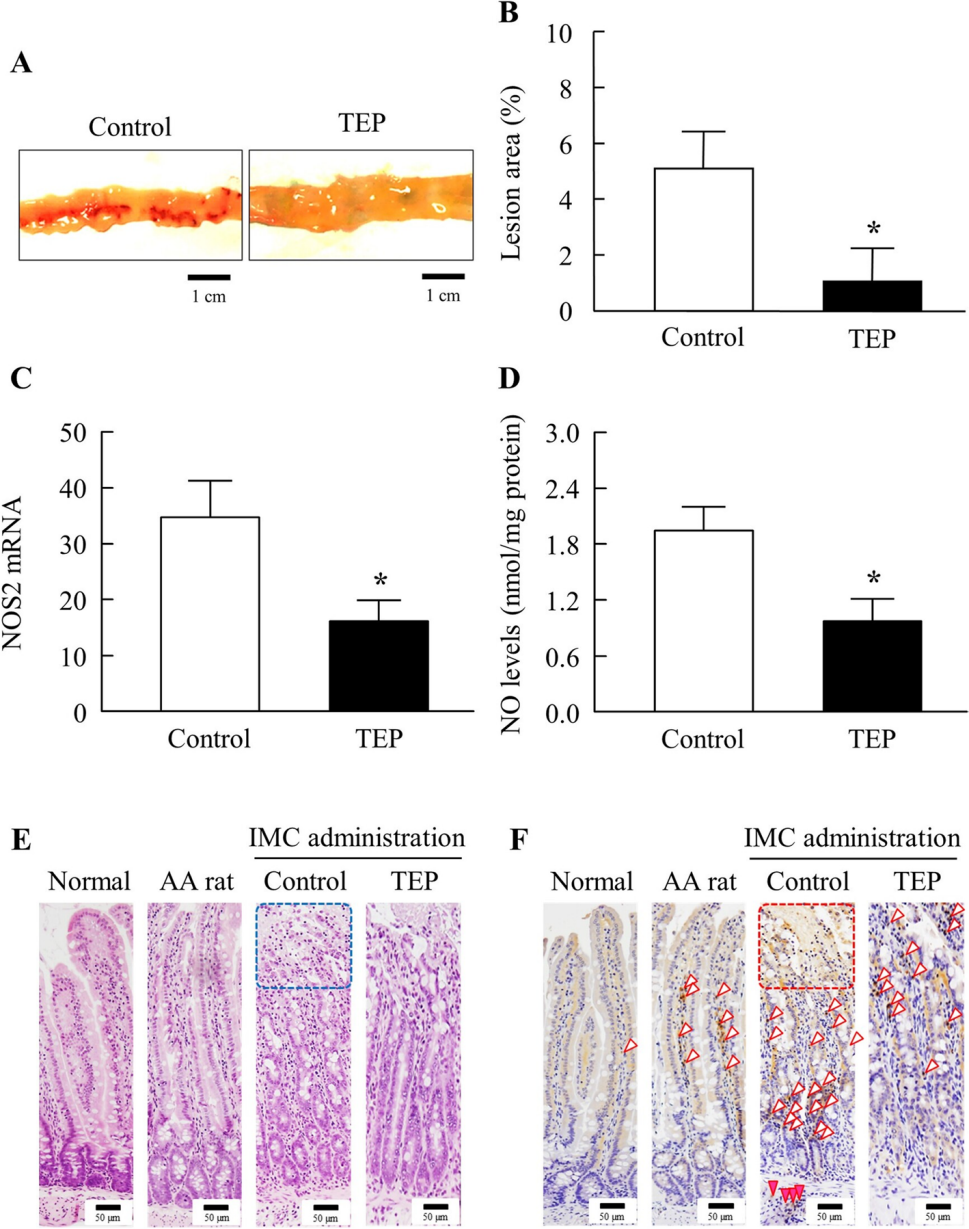
Effect of the TEP on IMC-Induced Jejunal Ulcerogenic Lesions in AA Rats 14 day after Adjuvant Injection. **A** images of the of IMC-treated AA rats treated with TEP. **B**–**D** effect of TEP on lesion area, NOS2 mRNA and NO levels in the jejunum of IMC-treated AA rats. **E** and **F** image of H&E-stained (**E**) and OPN immunostained jejunum tissue of IMC-treated AA rats treated with TEP. Squares, the tips of the intestinal villi in the mucosa of without TEP. Open arrowheads, OPN immunostained-positive cells in the mucosa. Closed arrowheads, OPN immunostained-positive cells in the mucosal myotome. Bars in the image indicate 50 µm. *n*=3–9. *P<0.05 vs control for each category. TEP attenuated the excessive NO *via* NOS2 levels, and promote recovery from IMC-induced jejunal ulcerogenic lesions in AA rats

**Fig. 6:**
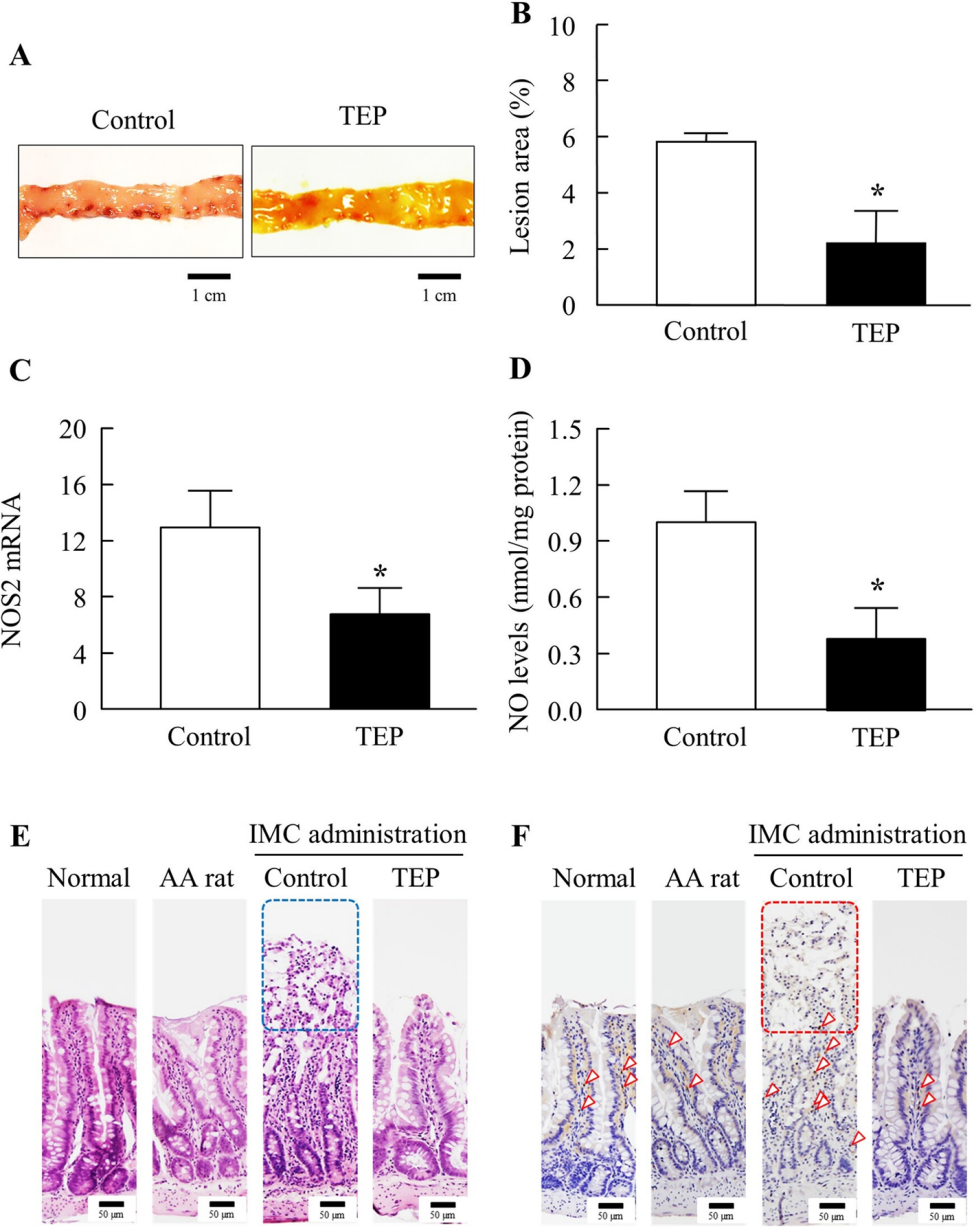
Effect of the TEP on IMC-Induced Ileal Ulcerogenic Lesions in AA Rats 14 day after Adjuvant Injection. **A** images of the ileum of IMC-treated AA rats treated with TEP. **B**–**D**:effect of TEP on lesion area, NOS2 mRNA and NO levels in the ileum of IMC-treated AA rats. **E** and **F** image of H&E-stained (**E**) and OPN immunostained ileum tissue of IMC-treated AA rats treated with TEP. Squares, the tips of the intestinal villi in the mucosa of without TEP. Open arrowheads, OPN immunostained-positive cells in the mucosa. Bars in the image indicate 50 µm. *n*=3–9. *P<0.05 vs control for each category. TEP attenuated the excessive NO *via* NOS2 levels, and promote recovery from IMC-induced ileal ulcerogenic lesions in AA rats

Figures 5 and 6 show the changes in jejunal and ileal lesions in IMC-treated AA rats, with or without TEP treatment. Similar to the gastric ulcerogenic lesions, TEP treatment promoted the recovery of ulcerogenic lesions in the jejunum and ileum compared to IMC-treated AA rats without TEP treatment (control group). In addition, the NOS2 mRNA and NO levels in the jejunum and ileum of IMC-treated AA rats were significantly lower than those in the control group. Moreover, NOS2 mRNA levels in normal rats were 0.36 ± 0.14, 0.018 ± 0.036 in the jejunum and ileum, and those in AA rats were 0.91 ± 0.45, and 0.025 ± 0.055 in the jejunum and ileum, respectively. NO levels in normal rats were 0.18 ± 0.073 (nmol/mg protein) and 0.018 ± 0.0091 (nmol/mg protein) in the jejunum and ileum, and those in AA rats were 0.29 ± 0.13 (nmol/mg protein) and 0.11 ± 0.012 (nmol/mg protein) (*n*=6), respectively. H&E-stained tissue specimens showed that TEP-treated AA rats were slightly affected by IMC, although their mucosal epithelium was, they were less inflamed than that of the AA rats. However, in the control group, dissolution of the tips of intestinal villi in the mucosa was observed in some areas. OPN immunostaining of the jejunum showed OPN-positive cells in the central part of the intestinal villi of the mucosa in the normal, AA, and TEP rats. In contrast, in the control group, OPN-positive cells were mostly observed at the bottom of the intestinal villi, and some cells were positive in the mucosal muscle plate. Immunostaining of the ileum revealed almost the same number of OPN OPN-positive cells. OPN-positive cells were also observed in the area where the tips of the intestinal villi in the mucosa of the control group mucosa were dissolved.

## Discussion

Although NSAIDs are essential in the treatment of RA, patients with RA are more likely to experience gastrointestinal injuries *via* NSAIDs compared to patients without RA. These side effects affect the RA treatment. In this study, we investigated the effect of TEP on IMC-induced gastrointestinal injuries induced by IMC using AA rats and found that oral administration of TEP promoted the repair of IMC-induced gastrointestinal injuries in this RA model.

It is important to select an appropriate animal model to examine the effects of TEP on NSAID-induced gastrointestinal injury associated with RA. Collagen-induced arthritis, AA, and streptococcal cell wall-induced arthritis models are used as rodent models of RA [17, 18]. AA develops and rapidly progresses into polyarthritis. In the early stages of inflammation, interleukin 17 (IL-17), interferon, and tumor necrosis factor α, which are involved in macrophage activation, are expressed. In the later stages of inflammation, the levels of IL-4, IL-6, and transforming growth factor-β increase. Consequently, AA exhibits symptoms similar to human RA, such as joint swelling, cartilage deterioration, and lymphocyte damage [17, 18]. Therefore, it is often used as an animal model of RA. Moreover, in an RA model, gastrointestinal injuries were observed in normal rats after IMC administration, although they were more pronounced in AA rats [19]. Based on these reports, here we used AA rats instead of normal rats.

In this study, paw edema was confirmed 7 days after adjuvant administration in rats. Paw edema peaked on day 14 (Fig. 2). Paw edema was also observed after 42 days; however, the lesion areas in the stomach, jejunum, and ileum were significantly worse after 14 days (Fig. 3). When IMC was administered to normal rats, the lesion area (%) to the stomach, jejunum, and ileum was 0.56 ± 0.29, 0.56 ± 0.18, and 0.51 ± 0.15, respectively (*n*=6–15). Based on these results, we focused on the therapeutic effects of TEP on IMC-induced ulcers in AA rats. AA is a model of systemic inflammation, and the occurrence of paw edema in unadministered paws indicates the onset of systemic inflammation [7]. Gastrointestinal injury increases when systemic inflammation was caused [20]. In AA rats, systemic inflammation of AA rats at 14 days after adjuvant injection tended to increase more than at 21 and 42 days. In AA rats, local inflammation occurred 7 days after adjuvant injection, and systemic inflammation occurred after 14 days. Moreover, systemic inflammation peaked 14 days after adjuvant injection and decreased over time. Kato et al. reported that the onset of gastrointestinal injury caused by IMC occurs when systemic inflammation is enhanced [21]. IMC-induced gastrointestinal injuries were observed in AA rats on days 21 and 42 after adjuvant injection, and these injuries were similar to those observed in the control rats (IMC-induced normal rats) in this study. Therefore, in this study, the healing effect of TEP on IMC-induced gastric lesions was investigated in AA rats that experienced systemic inflammation 14 days after adjuvant administration. Paw edema of AA rats were similar in all groups.

OPN is an extracellular matrix protein [22] that is involved in biological processes such as bone remodeling, innate immunity, acute and chronic inflammation, and cancer [23, 24]. OPN expression is observed in immune cells such as intestinal epithelial cells, macrophages, dendritic cells, and T lymphocytes [25], suggesting an important role in acute and chronic inflammation [26]. OPN is expressed by various inflammatory cells and has been proposed as a biomarker for inflammatory diseases [24]. Therefore, in this study, we evaluated the therapeutic effects of TEP on IMC-induced gastrointestinal bleeding using digital imaging and immunohistochemical staining for OPN. Inflammation was defined as the presence of numerous OPN-positive cells. TEP administration resulted in fewer open arrows, indicating fewer OPN-positive cells, and reduced inflammation. Gastrointestinal injuries in normal rats were less severe than those in AA rats (Fig. 3). Our previous study showed that gastrointestinal injuries caused by IMC were similar in normal and sham-operated rats [9]. Analysis of the lesion area showed a significant decrease in the disability rate in the TEP-treated AA rats compared to that in the control group. Although, the HPβCD was used in combination with TEP to enhance the therapeutic effect of TEP, the efficacy did not increase in this study, since the lesion area (%) in the stomach, jejunum and ileum of IMC-induced AA rats treated with TEP alone (without HPβCD) was 3.8 ± 2.1, 1.6 ± 1.1, 2.5 ± 1.8%, respectively (*n*=3). Moreover, we previously reported that the lesion areas in IMC-induced AA rats treated with HPβCD were similar to those in IMC-induced AA rats [9]. These results suggest that HPβCD does not affect the therapeutic effects of TEP in rats with IMC-induced AA. However, it is unclear whether TEP is encapsulated by HPβCD. Therefore, further research on this topic is required. In addition, the oral administration of TEP significantly reduced NOS2 mRNA and NO levels in the stomachs of IMC-treated AA rats (Fig. 4). Similar results were obtained for the jejunum and ileum (Figs. 5 and 6). NO is abundant in the vascular endothelial cells and plays an important role in the regulation of blood flow. In addition, it is involved in the regulation of mucosal blood flow and the maintenance of mucus secretion in the small intestinal mucosa. However, excessive production of NOS2, an inducible isozyme, causes gastric and intestinal injuries [27]. Thus, the oral administration of TEP suppressed the factors involved in gastrointestinal injury. Thus, our study is the first to investigate the treatment of IMC-induced gastrointestinal injuries with TEP in AA rats, providing important information for the treatment of gastrointestinal injuries using NSAIDs in RA patients. On the other hand, PPIs are clinically used to treat gastrointestinal injuries in patients with RA and NSAIDs [8]. It remains to be determined whether PPIs or TEP are superior, and we believe that it is important to compare the therapeutic effects of PPIs and TEP, alone and in combination (Table 1).

**Table 1:** Therapeutic effect of the RBM and TEP on IMC-Induced gastrointestinal injuries in AA rats

Leasion area (%)	Stomach	Jejunum	Ileum
RBM	8.0 ± 1.5	5.0 ± 1.4	4.9 ± 1.3
TEP	2.0 ± 0.8*	1.1 ± 1.2*	2.3 ± 1.3*

The RBM data have been previously reported [9] and Table 1 shows data that was remeasured to compare the therapeutic effects of TEP and RBM on gastrointestinal injuries in AA rats

^*^P<0.05 vs. RBM for each category

In clinical practice, TEP is administered at a dose of 50–150 mg/day. In this study, we estimated that the average human body weight was approximately 50 kg, and the dose of TEP was determined to be 150 mg/50 kg (3 mg/kg). The RBM dose (2 mg/kg) was determined based on the same concept used for TEP. On the other hand, package inserts have shown that the recovery effect of TEP on acetic acid-induced ulcers increases with increasing concentration [28]. Moreover, Katoh et al. investigated the inhibitory activities of TEP (3.2–200 mg/kg, oral administration) on taurocholic acid/ HCl induced acute gastric mucosal lesions in rats, and showed that the lesion levels (%) were approximately 80% in the rat treated with 3.2 and 12.5 mg/kg of TEP [29]. In addition, the lesion levels (%) decreased to approximately 46% with 50 and 200 mg/kg TEP [29]. Previous studies have shown that the inhibitory activity of TEP is dose-dependent. In this study, we determined the dose of TEP based on the human dosage, and the dose (3 mg/kg) was lower than that reported in previous reports (100–200 mg/kg). Therefore, high doses of TEP may increase recovery from IMC-induced gastrointestinal injury in AA rats. However, the RBM dose was not within the upper limit. From these results, the RBM dosage can be increased, which may change the results when compared to TEP. That is a subject for future research.

We also discussed the mechanisms for the recovery effects of TEP on IMC-induced ulcers in the AA rat model. It has been reported that TEP induces and enhances the expression of heat shock protein (HSP) 70, suppresses the production of pro-inflammatory cytokines and NO, and induces HSP to inhibit NOS2 [30, 31]. Therefore, these factors are thought to improve NOS2 mRNA and NO levels and influence healing. On the other hand, it also suggests the involvement of increased eNOS expression [32, 33]. Although eNOS has also been reported to increase in an IMC-induced model of gastrointestinal injury [34], Kato et al. reported that NOS2 increased more than eNOS in an IMC-induced gastrointestinal injury model [34]. These findings suggest that NOS2 is more closely related to IMC-induced gastrointestinal bleeding than eNOS. RBM has also been reported to inhibit enhanced NO production *via* NOS2 activity induced by excess IMC [35]. This study confirmed that both drugs suppressed NOS2 mRNA and NO levels, which are thought to affect the healing of gastrointestinal injuries. Moreover, TEP protects the gastric mucosa by increasing the production of PGs [36], which has also been observed in RBM [37]. This suggests that a different factor underlies the effect of TEP in the treatment of gastrointestinal injuries *via* NSAIDs in AA rats. The secretion of bicarbonate ions (HCO^3-^) in the duodenum is reduced by IMC and inhibits PG production [38, 39]. HCO^3-^ is secreted by secretin after TEP administration [40]. However, these effects were not observed with the RBM. Therefore, this factor may have a greater effects on the healing effects of TEP. On the other hand, many studies on the mechanism of NSAID-induced gastrointestinal injury in AA rats have been reported [41]. However, the exact mechanism remains unclear, and the difference in the inhibitory mechanism of TEP between AA rats and normal rats has not been fully elucidated. Further studies are needed to clarify the relationship between TEP and HCO^3-^ in gastrointestinal injuries in AA rats.

Furthermore, we compared the effects of TEP on IMC-treated normal rats with those on IMC-treated AA rats. In this study, gastrointestinal injuries were not detected in IMC-treated normal rats when TEP was administered (N.D., *n*=3). Thus, TEP is effective in treating IMC-induced gastrointestinal injuries in both normal and AA rats and may be more effective in serious gastrointestinal injuries of RA induced by NSAIDs.

IMC, which is likely to cause gastrointestinal disturbances, was used in this study. IMC-induced gastrointestinal injury models have been used in other experiments [30]. However, it is also important to elucidate the therapeutic effect of TEP on gastrointestinal injuries induced in AA rats administered COX-2 selective inhibition such as celecoxib. Therefore, we investigated the efficacy of TEP treatment for gastrointestinal injuries induced by different NSAIDs in AA rats. In addition, we plan to consider inflammatory biomarkers in future studies.

## Conclusions

We found that TEP was effective in the treatment of gastrointestinal injuries *via* NSAIDs in an RA model, and showed that TEP may be useful and provide many advantages in the treatment of serious intestinal lesions caused by NSAIDs in patients with RA. These findings provide significant data that can be used to design further studies aimed at developing drugs to treat gastrointestinal injuries *via* NSAIDs in patients with RA.

## Data Availability

All data generated in this study may be requested from the corresponding author.

## Abbreviations

GAPDH: Glyceraldehyde-3-phosophate
H&E: Hematoxylin and eosin
HPβCD: 2-hydroxypropyl-β-cyclodextrin
HSP: Heat shock protein
IL: Interleukin
IMC: Indomethacin
iNOS, NOS2: Inducible NO synthase
NO: Nitric oxide
NSAIDs: Nonsteroidal anti-inflammatory drugs
OPN: Osteopontin
PG: Prostaglandin
PPI: Proton pump inhibitor
RA: Rheumatoid arthritis
RBM: Rebamipide
S.E.: Standard error
TEP: Teprenone
